# Diverse effects of prostacyclin on angiogenesis-related processes in the porcine endometrium

**DOI:** 10.1038/s41598-023-41197-z

**Published:** 2023-08-29

**Authors:** Magdalena Szymanska, Agnieszka Blitek

**Affiliations:** Institute of Animal Reproduction and Food Research of the Polish Academy of Sciences, Tuwima 10, 10-748 Olsztyn, Poland

**Keywords:** Cell biology, Molecular biology

## Abstract

Angiogenesis is important for endometrial remodeling in mature females. The endometrium synthesizes high amounts of prostacyclin (PGI2) but the role of PGI2 in angiogenesis-related events in this tissue was not fully described. In the present study, porcine endometrial endothelial (pEETH) cells and/or a swine umbilical vein endothelial cell line (G1410 cells) were used to determine the regulation of PGI2 synthesis and PGI2 receptor (PTGIR) expression by cytokines and to evaluate the effect of PGI2 on pro-angiogenic gene expression, intracellular signaling activation, cell proliferation and migration, cell cycle distribution, and capillary-like structure formation. We found that IL1β, IFNγ, and/or TNFα increased PGI2 secretion and *PTGIR* expression in pEETH cells. Iloprost (a PGI2 analogue) acting through PTGIR enhanced the transcript abundance of *KDR*, *FGFR2*, and *ANGPT2* and increased proliferation of pEETH cells. This latter was mediated by PI3K and mTOR activation. In support, transfection of G1410 cells with siRNA targeting PGI2 synthase decreased pro-angiogenic gene expression and cell proliferation. Furthermore, iloprost accelerated the gap closure and promoted cell cycle progression. Intriguingly, the formation of capillary-like structures was inhibited but not completely blocked by iloprost. These findings point to a complex pleiotropic role of PGI2 in angiogenesis-related events in the porcine uterus.

## Introduction

Angiogenesis involves a series of steps starting with the activation of endothelial cells, their migration towards the angiogenic stimulus, proliferation, tube formation, and vessel stabilization^1, 2^. Numerous factors are involved in the regulation of these processes, including vascular endothelial growth factor (VEGF), fibroblast growth factor (FGF), angiopoietins (ANGPTs) and their receptors^2–4^. Angiogenesis is not restricted to embryonic development but also takes place in adults, where it is observed in both physiological and pathological processes. In the female reproductive tract, intense angiogenesis was demonstrated during corpus luteum formation^5^ and endometrial remodeling^2^.

The endometrium is one of the most dynamic tissues in the organism and undergoes cyclical growth and regression throughout the female reproductive life. This tissue undergoes morphological and physiological changes during each menstrual or estrous cycle in order to establish the proper environment for embryo implantation. Although the overall control of endometrial remodeling is primarily governed by estrogen and progesterone^6, 7^, the role of ovarian steroids in endometrial angiogenesis is less clear^2^. It is likely that locally produced growth factors, cytokines, prostaglandins (PGs), and other biologically active molecules, that have been identified in the endometrium substantially regulate angiogenic events.

Prostaglandin I2 (PGI2), commonly known as prostacyclin, belongs to prostanoid family of lipid mediators, which are derivatives of arachidonic acid. Prostaglandin endoperoxide synthase catalyzes the conversion of arachidonic acid to unstable intermediate, PGH2. Afterward, a membrane-bound PGI2 synthase (PTGIS) rearranges PGH2 to form PGI2. PTGIS is mainly localized in endothelial and smooth muscle cells, but is also expressed in several other cell types in the organism^8, 9^. PGI2 plays an important role in the cardio-vascular system being a potent vasodilator and an inhibitor of platelet aggregation^10^ and has been reported as a mediator of protective effects of VEGF on the vascular system^11^. PGI2 may also be involved in pregnancy establishment and embryo implantation. Sustained overproduction of PGI2 in the uterus is physiologically evident during pregnancy in women^12^, mice^13^, and domestic species^14, 15^.

PGI2 exerts its biological action on target cells acting through a PGI2-specific G protein-coupled receptor, PTGIR. PTGIR is a typical seven transmembrane domain receptor, which upon PGI2 ligation causes an adenylate cyclase activation via Gs subunit resulting in a rapid cAMP formation^16^. This classical PGI2 signaling is involved in the modulation of vascular function and smooth muscle relaxation^8, 16^. Moreover, PGI2-PTGIR coupling may lead to phospholipase C activation and calcium mobilization^17^, probably via Gq subunit^16^.

An important role for PGI2-PTGIR signaling pathway in cyclical regeneration and regression undergone by the human endometrium has been suggested^18^. A greater expression of PTGIR in endometrial tissue was observed during the menstrual stage compared with proliferative and secretory stages of the cycle^19^. Moreover, the concentration of PGI2 in the venous blood was maximal during the menstruation^20^. Spatio-temporal expression of PTGIR has also been demonstrated in the endometrium of farm animals^14, 21^. As we previously reported^21^, PTGIR expression in luminal epithelial and stromal cells of the porcine endometrium increased in response to conceptus signals and the activation of PTGIR by iloprost, a stable analogue of PGI2, stimulated cAMP formation. Moreover, culture of uterine stromal cells with iloprost elevated the abundance of *VEGF* and *FGF2* transcripts. Similarly, iloprost induced the expression of *FGF*, *AGNPT1*, and *ANGPT2* in human endometrial biopsies in vitro^18^. These data indicate that PGI2 may promote angiogenesis in the uterine endometrium. The present study was conducted to verify this hypothesis, in particular to determine whether PGI2 and its receptor signaling affect angiogenesis-related processes in the uterus. We used endometrial endothelial cells of the pig to examine the regulation of PGI2 synthesis and PTGIR expression; the effect of PGI2 on the expression of angiogenesis- and vascular function-related factors, cell proliferation, and capillary-like structure formation; and to identify intracellular pathways involved in PGI2 action. To further verify the physiological relevance of PGI2 in angiogenesis-related processes, immortalized swine umbilical vein endothelial cells (SUVECs, G1410 cell line) were used to analyze the transcript abundance of angiogenesis-related factors, cell proliferation and migration, cell cycle progression, and the formation of capillary-like structures.

## Results

### PGI2 receptor expression in pEETH and G1410 cells

We first confirmed that cultured porcine endometrial endothelial (pEETH) cells and G1410 cells stained positively for a marker of endothelial cells, von Willebrand Factor (vWF). Moreover, pEETH and G1410 cells prominently expressed PTGIR protein in the cytoplasm and nuclear membranes (Supplementary Figs. S1 and S2). Thus, both cell types displayed similar characteristics and were used to examine the effect of PGI2 on angiogenesis-related processes.

### Effect of cytokines on PGI2 synthesis and PGI2 receptor expression in pEETH cells (Experiment 1)

**Figure 1:**
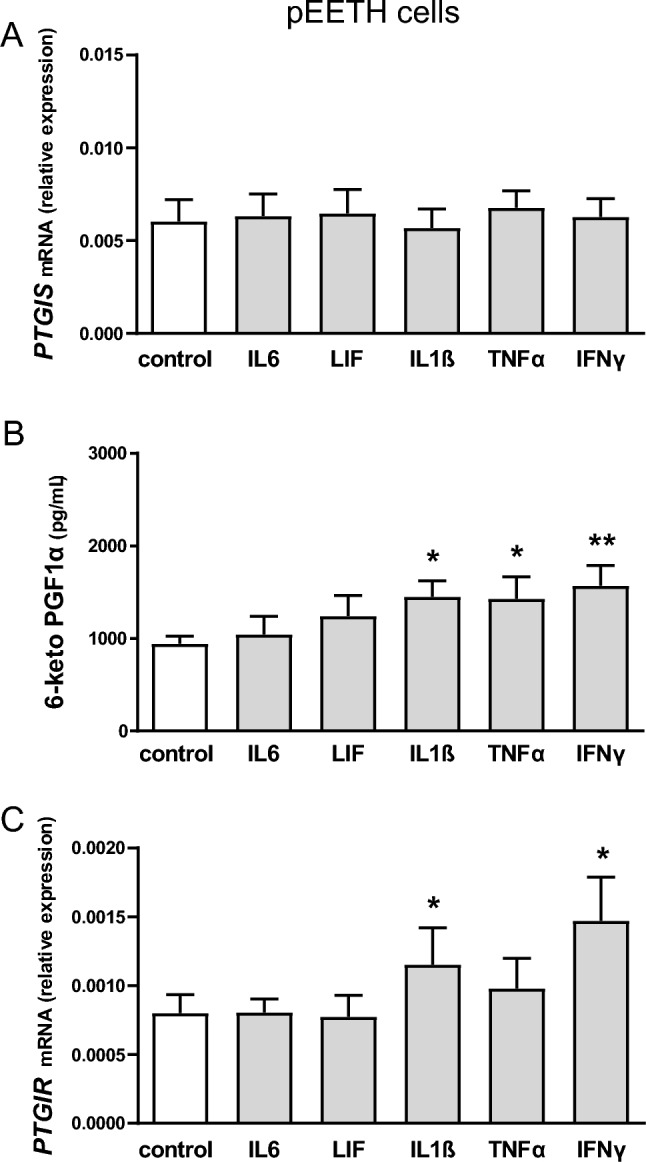
Cytokines affect prostacyclin (prostaglandin I2; PGI2) synthesis and PGI2 receptor (PTGIR) expression in porcine endometrial endothelial (pEETH) cells. Effect of interleukin 6 (IL6), leukemia inhibitory factor (LIF), interleukin 1β (IL1β), tumor necrosis factor α (TNFα), and interferon γ (IFNγ) on PGI2 synthase (*PTGIS*; **A**) and *PTGIR* (**C**) transcript abundance in pEETH cells, and 6-keto PGF1α (a PGI2 metabolite) concentrations in the incubation medium (**B**). Cells were exposed to cytokines (all at the concentration of 10 ng/mL) for 6 h. Data are expressed as the mean ± SEM (n = 6). Asterisks indicate differences as compared with the control cells (*p < 0.05; **p < 0.01).

None of the examined cytokines affected the expression of *PTGIS* mRNA in pEETH cells (Fig. 1). However, the concentration of 6-keto PGF1α (a stable metabolite of PGI2) in the incubation medium increased in the presence of interleukin 1β (IL1β; *p* < 0.05), tumor necrosis factor α (TNFα; *p* < 0.05) or interferon γ (IFNγ; *p* < 0.01) compared with non-treated cells. Furthermore, the addition of IL1β or IFNγ into pEETH cell culture resulted in the elevated abundance of *PTGIR* transcripts as compared with the control value (*p* < 0.05).

### Effect of iloprost on the transcript abundance of genes related to angiogenesis and vascular function in pEETH cells (Experiment 2)

**Figure 2:**
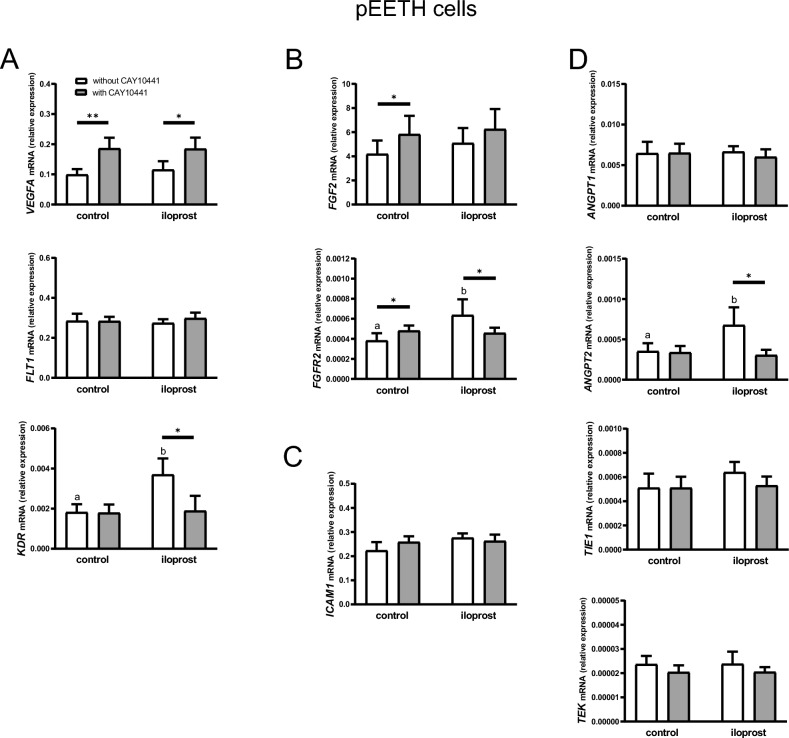
Prostacyclin (prostaglandin I2; PGI2) modulates the expression of angiogenesis-related factors in porcine endometrial endothelial (pEETH) cells. Effect of iloprost (a PGI2 analogue) on the mRNA expression of vascular endothelial growth factor (*VEGFA*) and its receptors (*FLT1* and *KDR*; **A**), fibroblast growth factor 2 (*FGF2*) and its receptor (*FGFR2*; **B**), intercellular adhesion molecule 1 (*ICAM1*; **C**), and angiopoietins (*ANGPT1* and *ANGPT2*) and their receptors (*TIE1* and *TEK*; **D**) in pEETH cells. Cells were pre-incubated with or without CAY10441 (a PTGIR antagonist; 10 µM) for 30 min, followed by the culture for 24 h without (control) or with iloprost (0.1 µM). Data are expressed as the mean ± SEM (n = 6). Mean values with various superscripts differ significantly (*p* ≤ 0.05; a and b for cells not exposed to CAY10441). Asterisks indicate differences among control or iloprost-treated cells (*p < 0.05; **p < 0.01).

As shown in Fig. 2, relative mRNA expression of *VEGFA*, kinase insert domain receptor (*KDR*; VEGF receptor 2), *FGF2*, FGF receptor (*FGFR2)*, and *ANGPT2*, but not fms related receptor tyrosine kinase 1 (*FLT1*; VEGF receptor 1), intercellular adhesion molecule 1 (*ICAM1*), *ANGPT1*, or ANGPT receptors (*TIE1* and *TEK*) in pEETH cells, was affected by iloprost (a stable analogue of PGI2) and/or CAY10441 (an antagonist of PTGIR) presence.

The abundance of *VEGFA* transcripts was affected by CAY10441 (*p* = 0.02), but not iloprost (*p* = 0.89) presence. Almost two-fold greater *VEGFA* mRNA expression was detected in pEETH cells exposed to CAY10441 both in the absence or presence of iloprost as compared with respective control values (*p* < 0.01 and *p* < 0.05).

Iloprost presence (*p* = 0.047) and iloprost by CAY10441 interaction (*p* = 0.03) influenced *KDR* mRNA expression in pEETH cells. The treatment of cells with iloprost stimulated the abundance of *KDR* transcripts (*p* < 0.05), and this effect was abolished by blocking PGI2 receptors with CAY10441 (*p* < 0.05).

The addition of a PTGIR inhibitor, CAY10441, to pEETH cells affected *FGF2* mRNA expression (*p* = 0.05). The transcript level of *FGF2* increased after the treatment of cells with CAY10441 and this effect was clearly visible in cells not exposed to iloprost (*p* < 0.05).

Iloprost presence and iloprost by CAY10441 interaction was detected in pEETH cells in regard to *FGFR2* mRNA expression (*p* = 0.05 and *p* = 0.04; respectively). The treatment of cells with iloprost increased the abundance of *FGFR2* transcripts as compared with non-treated control (*p* < 0.05). The addition of CAY10441 abolished the stimulatory effect of iloprost (*p* < 0.05). Moreover, CAY10441 alone elevated the mRNA expression of this receptor (*p* < 0.05).

The relative abundance of *ANGPT2* transcripts was affected by iloprost (*p* = 0.05) and iloprost by CAY10441 interaction (*p* = 0.04). The treatment of pEETH cells with iloprost resulted in an almost two-fold greater *ANGPT2* mRNA expression compared with non-treated cells (*p* < 0.05). The addition of CAY10441 significantly blocked the stimulatory effect of iloprost (*p* < 0.05).

### Effect of PTGIS silencing on the transcript abundance of selected genes related to angiogenesis and vascular function in G1410 cells (Experiment 3)

**Figure 3:**
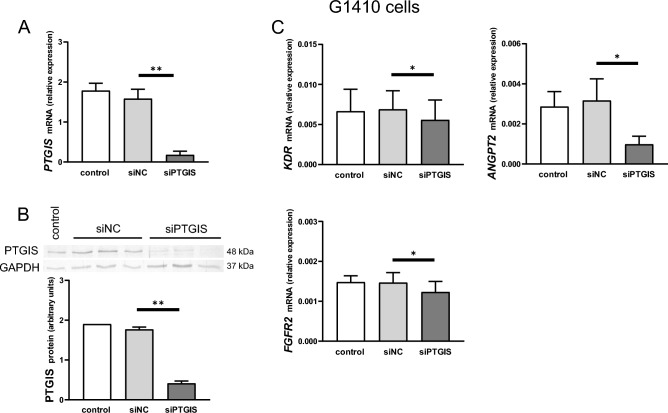
Prostaglandin I2 synthase (PTGIS) expression is important for the abundance of angiogenesis-related factors in endothelial cells. Effect of *PTGIS* silencing using siRNA on the mRNA (**A**) and protein (**B**) expression of PTGIS and the transcript abundance of vascular endothelial growth factor receptor (*KDR*), fibroblast growth factor receptor 2 (*FGFR2*), and angiopoietin 2 (*ANGPT2*; **C**) in immortalized swine umbilical vein endothelial cells (G1410 cells). Cells were transfected with siRNA targeting *PTGIS* (siPTGIS) or with scrambled siRNA (siNC), or incubated with Opti-MEM alone (control) for 6 h. Then, media were discarded and cells were cultured for 24 (for mRNA analysis) or 48 (for protein analysis) h in MCDB-131 medium supplemented with antibiotics and 1% newborn calf serum. Data are expressed as the mean ± SEM (n = 3–4). Asterisks indicate differences between siNC- and siPTGIS-transfected cells (*p < 0.05; **p < 0.01).

To confirm the *PTGIS* knock-down, PTGIS mRNA and protein expression were analyzed. The transfection of G1410 cells with small interfering RNA (siRNA) construct targeting *PTGIS* (siPTGIS) efficiently reduced PTGIS mRNA and protein expression by 90 and 75%, respectively (*p* < 0.01; Fig. 3A, B, Supplementary Fig. S3).

To verify the importance of endogenous PGI2 for endothelial cells, iloprost-stimulated genes from Experiment 2 were examined in G1410 cells with a knock-down of *PTGIS*. As shown in Fig. 3C, *PTGIS*-silenced G1410 cells expressed lower abundance of *KDR*, *FGFR2*, and *ANGPT2* transcripts as compared with scrambled siRNA (siNC)-transfected cells (*p* < 0.05).

### Effect of iloprost on pEETH cell proliferation (Experiment 4)

**Figure 4:**
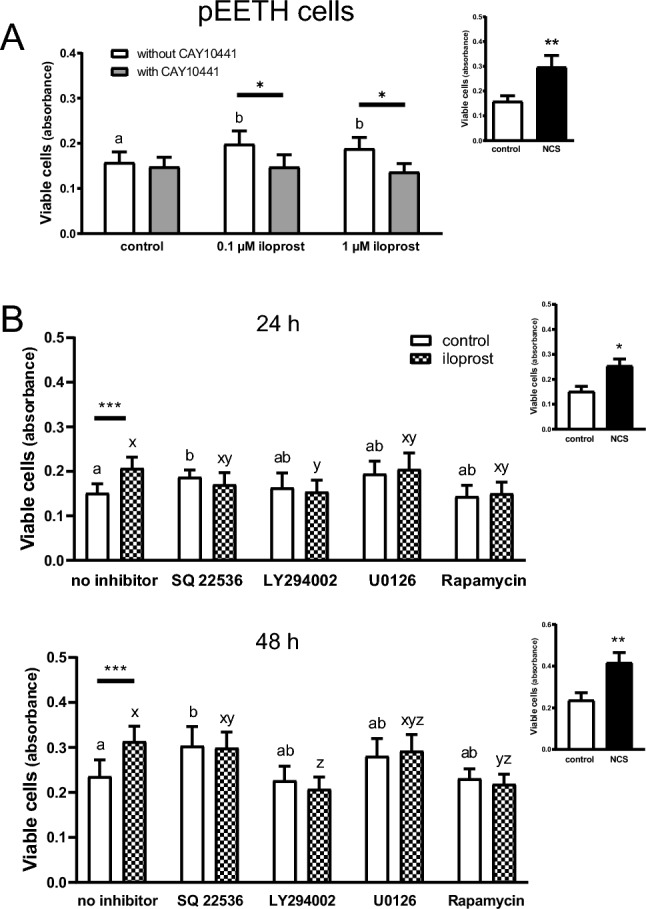
Prostacyclin (prostaglandin I2; PGI2) acting via its membrane receptor, PTGIR, and through the activation of phosphatidylinositol 3 kinase (PI3K) and mTOR pathway stimulates proliferation of porcine endometrial endothelial (pEETH) cells. The involvement of PTGIR in PGI2-affected pEETH cell proliferation (**A**). Cells were serum starved for 16 h, followed by the pre-incubation with or without CAY10441 (a PTGIR antagonist; 10 µM) for 30 min. Then, cells were cultured without (control) or with iloprost (a PGI2 analogue; 0.1 or 1 µM) in the presence or absence of CAY10441 for the next 24 h. Identification of intracellular pathways involved in PGI2-affected pEETH cell proliferation (**B**). After serum starving for 16 h, cells were pre-incubated without (no inhibitor) or with: adenylate cyclase inhibitor (SQ 22536; 10 µM), PI3K inhibitor (LY294002; 20 µM), MEK1 and MEK2 kinase inhibitor (U0126; 20 µM), or mTOR kinase inhibitor (rapamycin; 20 nM) for 1 h. Then, cells were treated without (control) or with iloprost (0.1 µM) for 24 or 48 h. Newborn calf serum (NCS; small insets) was used as a positive control. All data are expressed as the mean ± SEM (n = 6). Mean values with various superscripts differ significantly (*p* < 0.05; a and b for cells not exposed to iloprost; x, y, and z for cells exposed to iloprost). Asterisks indicate differences among particular type of treatment (*p < 0.05; **p < 0.01; ***p < 0.001).

The number of viable cells was affected by iloprost (*p* = 0.03) and by CAY10441 (*p* = 0.001; Fig. 4A). Both doses of iloprost stimulated the proliferation of pEETH cells compared with non-treated cells (26% and 19% increases for 0.1 and 1 µM iloprost, respectively; *p* < 0.05) after 24 h. Moreover, the addition of CAY10441 abolished the stimulatory effect of iloprost (*p* < 0.05). The culture of pEETH cells in the presence of newborn calf serum (NCS), used as a positive control, increased the number of viable cells as compared with control cells (88% increase; *p* < 0.01).

Based on results presented in Fig. 4A, a dose of 0.1 µM iloprost was chosen to identify the intracellular pathways involved in iloprost-stimulated pEETH cell proliferation. Both the treatment of cells with iloprost and the presence of a respective kinase inhibitor affected cell proliferation (Fig. 4B). Iloprost increased the number of viable cells after 24 and 48 h of culture (*p* < 0.001). The addition of LY294002 (an inhibitor of PI3K) abolished the stimulatory effect of iloprost on the proliferation after both 24 and 48 h (*p* < 0.05 and *p* < 0.01; respectively). Moreover, an inhibitor of mTOR kinase, rapamycin, blocked the proliferative capacity of pEETH cells exposed to iloprost for 48 h (*p* < 0.05). Basal, not iloprost-stimulated cell proliferation was greater after 24 and 48 h in the presence of SQ 22536 (an adenylate cyclase inhibitor) as compared with the respective control cells (*p* < 0.05). NCS increased the number of viable pEETH cells after both time periods (*p* < 0.05).

### Effect of iloprost on proliferation and cell cycle progression in G1410 cells (Experiments 5 and 6)

**Figure 5:**
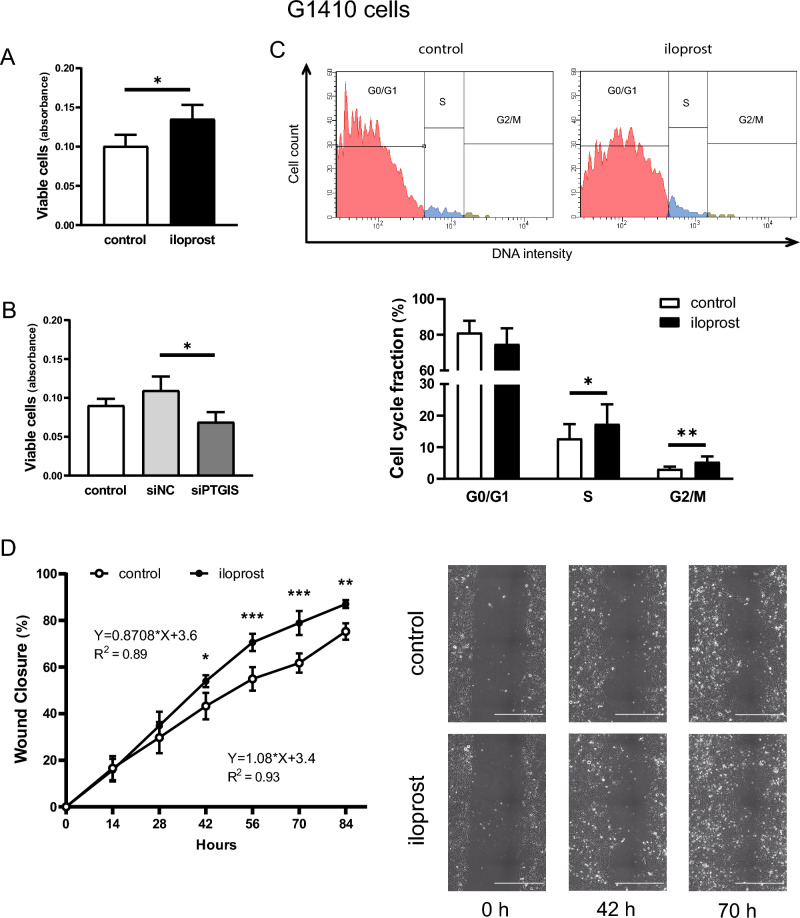
Prostacyclin (prostaglandin I2; PGI2) stimulates endothelial cell proliferation rather than their migration and promotes cell cycle progression. Effect of iloprost (a PGI2 analogue) on immortalized swine umbilical vein endothelial cell (G1410 cells) proliferation (**A**), cell cycle progression (**C**), and cell migration (**D**) and the consequences of PGI2 synthase (*PTGIS)* silencing using siRNA on G1410 cell proliferation (**B**). G1410 cells were exposed to medium only (control) or medium containing iloprost (0.1 µM) for 48 h followed by the analyses of the number of viable cells using CellTiter 96 Aqueous One Solution Reagent (**A**) and the percentage of cells in G0/G1, S and G2/M phases were calculated by fluorescence intensity of incorporated FxCycle Violet Stain in a flow cytometry analysis (**C**). Moreover, G1410 cells were transfected with siRNA targeting *PTGIS* (siPTGIS), or with scrambled siRNA (siNC), or incubated with Opti-MEM alone for 6 h. Then, media were changed and cells were cultured for 48 h and the number of viable cells was determined using CellTiter 96 Aqueous One Solution Reagent (**B**). The wound healing assay was performed to examine cell migration (**D**). To this end, G1410 cells were cultured in the Culture-Insert 2 Well until reaching 100% confluence. After removing the Culture-Inserts, cells were treated without (control; open dots) or with iloprost (0.1 µM; black dots) and monitored for 84 h. The area of the gap was measured (**D**). All data are expressed as the mean ± SEM (n = 3 in **A**–**C** or n = 4 in **D**). Asterisks indicate differences between siNC and siPTGIS-transfected cells and between control and iloprost-treated cells (*p < 0.05; **p < 0.01; ***p < 0.001). Representative images of cell cycle fractions (**C**) and the wound closure at time lapse 0, 42, and 70 h (**D**) are presented; scale bars 500 µm.

Similarly to pEETH cells, G1410 cells responded to iloprost treatment with increased cell proliferation; the addition of a PGI2 analogue stimulated the number of viable G1410 cells as compared with the control value (34% increase; *p* < 0.01; Fig. 5A). Furthermore, limiting endogenous PGI2 synthesis by *PTGIS* silencing inhibited G1410 cell proliferation as compared with cells transfected with siNC (36% decrease; *p* < 0.05; Fig. 5B).

Figure 5C shows results of the cell cycle analysis of G1410 cells. There was a difference in the number of cells between each phase of the cell cycle in both control and iloprost-treated cells (*p* < 0.001). Iloprost increased the proportion of cells in S (from 12.8 to 17.4%; *p* < 0.05) and G2/M (from 3.1 to 5.4%; *p* < 0.01) phases as compared with control cells.

### Effect of iloprost on G1410 cell migration (Experiment 7)

The wound-healing assay was used to examine the effect of iloprost on migration of G1410 cells. As demonstrated in Fig. 5D, the wound closure was affected by time of culture (*p* < 0.0001) and proceeded during examined period for both the control and iloprost-treated cells (R^2^ = 0.93 and R^2^ = 0.89, respectively). The treatment of G1410 cells with iloprost stimulated the wound closure as compared with non-treated cells and this effect was visible at the following time points: 42 (53.9 vs. 43.2%; *p* < 0.05), 56 (70.6 vs. 54.9%; *p* < 0.001), 70 (78.9 vs. 61.7%; *p* < 0.001), and 84 (87 vs. 75.3%; *p* < 0.01) h.

### Effect of iloprost on the formation of capillary-like structures by pEETH and G1410 cells (Experiment 8)

**Figure 6:**
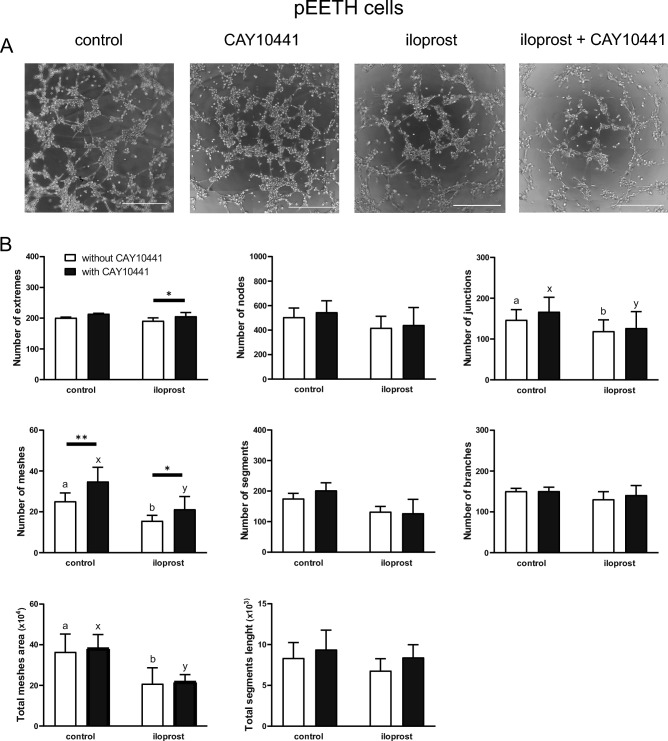
Prostacyclin (prostaglandin I2; PGI2) inhibits the formation of capillary-like structures by porcine endometrial endothelial (pEETH) cells. Cells were pre-incubated with or without CAY10441 (a PTGIR antagonist; 10 µM) for 30 min, followed by the incubation on µ-Slides angiogenesis plates without (control) or with iloprost (a PGI2 analogue; 0.1 µM) in the presence or absence of CAY10441. The number of extremes, the number of meshes, the total meshes area, the number of nodes, the number of segments, the total segments length, the number of junctions, and the number of branches were evaluated (**B**). Data are presented as the mean ± SEM (n = 3). Mean values with various superscripts differ significantly ( *p* ≤ 0.05; a and b for cells not exposed to CAY10441, x and y for cells exposed to CAY10441). Asterisks indicate differences among control or iloprost-treated cells (*p < 0.05; **p < 0.01). Representative images of capillary-like structures formed by pEETH cells are presented (**A**); scale bars 500 µm.

The formation of capillary-like structures by pEETH cells was affected by iloprost and/or the presence of a PTGIR antagonist, CAY10441 (Fig. 6). The number of junctions (*p* < 0.01), the number of meshes (*p* < 0.01), and total meshes area (*p* < 0.05) were lower after the treatment of cells with iloprost and these effects were observed both in the presence and absence of CAY10441. The addition of CAY10441 alone increased the number of meshes (*p* < 0.01). The presence of a PTGIR inhibitor (*p* < 0.05) increased the number of extremes and the number of meshes in iloprost-treated cells (*p* < 0.05).

**Figure 7:**
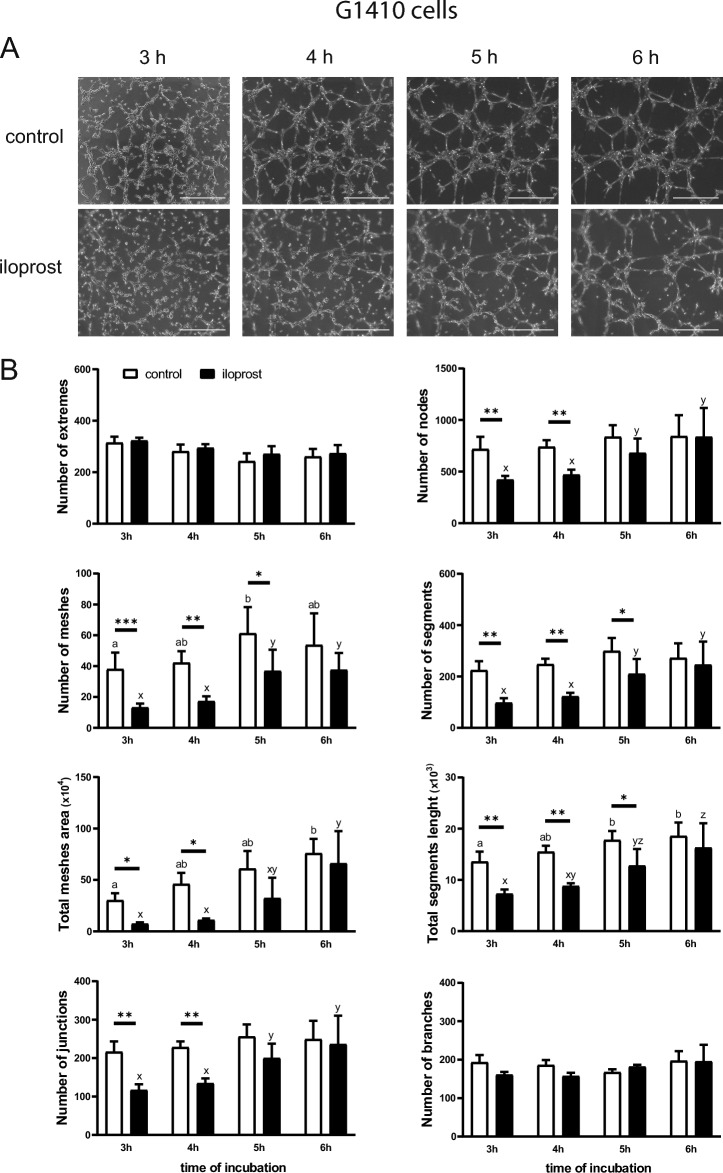
Prostacyclin (prostaglandin I2; PGI2) delays but does not completely block the formation of capillary-like structures by endothelial cells. Immortalized swine umbilical vein endothelial cells (G1410 cells) were incubated on µ-Slides angiogenesis plates without (control) or with iloprost (a PGI2 analogue; 0.1 µM). The number of extremes, the number of meshes, the total meshes area, the number of junctions, the number of nodes, the number of segments, the total segments length, and the number of branches after 3, 4, 5, and 6 h were evaluated (**B**). Data are presented as the mean ± SEM (n = 3). Mean values with various superscripts differ significantly (*p* ≤ 0.05; a and b for cells not exposed to iloprost, x, y, and z for cells exposed to iloprost). Asterisks indicate differences between control or iloprost-treated cells at particular time point (*p < 0.05; **p < 0.01; ***p < 0.001). Representative images of capillary-like structures formed by G1410 cells are presented (**A**); scale bars 500 µm.

To verify the surprising inhibitory effect of iloprost on the network formation, G1410 cells were also incubated with iloprost and the same parameters as described for pEETH cells were examined. The presence of iloprost and/or the time of incubation affected the number of nodes, junctions, meshes, and segments formed by these cells, and influenced the total meshes area and total segments length (*p* ≤ 0.05; Fig. 7). The addition of iloprost resulted in a smaller total meshes area (*p* < 0.05) and lower numbers of nodes and junctions (*p* < 0.01) observed after 3 and 4 h of incubation. An inhibitory effect of iloprost on the number of meshes, the number of segments and the total segments length was detected after 3, 4, and 5 h of treatment (*p* < 0.05). Of interest, none of the studied parameters differed between control and iloprost-treated G1410 cells after 6 h. Moreover, the numbers of meshes, junctions, nodes, and segments were greater in G1410 cells exposed to iloprost for 5 and 6 h as compared with 3 h of treatment (*p* ≤ 0.05). Total meshes area and total segments length increased between 3 and 6 h of incubation in both control and iloprost-treated cells (*p* < 0.05).

## Discussion

Angiogenesis and the proper vascular integrity contribute substantially to cyclical endometrial remodeling during the estrous cycle as well as to increased uterine blood flow during pregnancy^2, 22^. The porcine endometrium synthesizes high amounts of PGI2^15^ and PGI2-receptor system has been demonstrated as an important component of embryo-maternal interactions required for pregnancy establishment^21, 23^. This report showed that cytokines may increase the abundance of endometrial PGI2 and up-regulate the expression of PGI2 receptors. Moreover, we evidenced diverse physiologically relevant role of PGI2 in the porcine endothelial cells. PGI2 acting through its membrane receptor, PTGIR, and/or activating PI3 and mTOR kinases stimulated pEETH cell proliferation and increased the expression of pro-angiogenic factors in these cells. By contrast, PGI2 inhibited the formation of capillary-like structures by pEETH cells. Using G1410 cell line we demonstrated that PGI2 promoted cell cycle progression, stimulated cell proliferation rather than cell migration, and inhibited but did not completely block the formation of capillary-like structures. Moreover, results of the present study showed that endogenous PGI2 synthesis is required for endothelial cell proliferation and pro-angiogenic gene expression indicating auto- and/or paracrine actions of PGI2. All these findings point to a complex pleiotropic role of PGI2 in angiogenesis-related events in the porcine uterus.

Cytokines are present in the porcine uterus during both the estrous cycle and early pregnancy regulating the secretory activity of endometrial cells^24–26^. Interestingly, abundant secretion of PGI2 from the endometrium^15^ coincides with elevated levels of cytokines in the uterus^25, 26^. Results of the present study showed, that PGI2 secretion and *PTGIR* mRNA expression in pEETH cells were stimulated by IL1β and IFNγ. Moreover, the accumulation of PGI2 in the incubation medium increased in response to TNFα. These findings are in line with our previous data showing a stimulatory effect of IL1β on PGI2 release from endometrial tissue^15^ and a stimulatory effect of both IL1β and IFNγ on *PTGIR* transcript abundance in luminal epithelial and stromal cells of the porcine endometrium^21^. TNFα, in turn, was shown to induce PGI2 synthesis in human vascular endothelial and smooth muscle cells^27, 28^. Moreover, IL1β and TNFα increased the release of PGI2 from smooth muscle cells of the rat pulmonary artery^29^. Thus, elevated PGI2 secretion and *PTGIR* mRNA expression in endometrial endothelial cells in response to IL1β, IFNγ, or TNFα demonstrated here points to the regulatory role of cytokines in the development and/or function of the vascular system in the porcine uterus.

To evaluate the importance of abundant levels of PGI2 in the endometrium, the role of PGI2 in angiogenesis-related processes was further verified. First, we investigated the effect of PGI2 on the expression of factors involved in angiogenesis and/or controlling vascular functions. VEGF and its receptors, Flt1 and KDR, as well as FGF2 and its receptor, FGFR2, play the crucial role during angiogenesis modulating endothelial cell survival, proliferation, migration, and tube formation^4, 11^. Present results demonstrated that iloprost had no effect on the abundance of *VEGFA* and *FGF2* transcripts in pEETH cells but increased the mRNA expression of their receptors, *KDR* and *FGFR2*. This result seems surprising in light of our previous data showing a stimulatory effect of iloprost on the abundance of *VEGF* and *FGF2* transcripts in stromal cells^21^. Moreover, perfusion of lungs with PGI2 induced VEGF synthesis in rats^30^. On the other hand, iloprost induced *FGF2* mRNA expression in epithelial cells of the human^18^ but not porcine^21^ endometrium. In human peridontal ligament cells, in turn, amounts of *VEGF* but not *FGF2* transcripts were elevated by iloprost^31^. Thus, the modulatory effect of PGI2 on the abundance of *VEGF* and *FGF2* transcripts may be species and/or cell type specific. Nevertheless, *KDR* and *FGFR2* mRNA expression increased in porcine endometrial endothelial cells in response to a PGI2 analogue. It points to the possible role of PGI2 in the up-regulation of angiogenic receptors to potentiate VEGF and/or FGF2 action on endothelial cells.

In addition to VEGF and FGF, endothelial cell functions may also be affected by angiopoietins and ICAM. Angiopoietins acting via Tie1 or Tie2 receptors (the latter is encoded by *TEK* gene) participate in the maturation and stabilization of blood vessels and affect cell survival^5, 32^, while ICAM1 being an adhesion molecule enables cell–cell interaction and its role in angiogenesis was previously described^33^. As we demonstrated here, iloprost increased the mRNA expression of *ANGPT2* but not *ANGPT1* or its receptors or *ICAM1* in endometrial endothelial cells. ANGPT2 may promote angiogenesis inducing both endothelial cell migration and tube formation^32, 34^. Thus, ANGPT2 may be another factor important for endothelial cell activity that is modulated by PGI2 in the porcine uterus.

The stimulatory effect of a PGI2 analogue on the expression of *KDR*, *FGFR2*, and *ANGPT2* mRNA observed in the present study was abolished by the addition of a PTGIR antagonist, CAY10441. It indicates that PGI2 acts through its membrane receptor to induce the expression of these angiogenic factors in pEETH cells. Such result is in line with previously demonstrated importance of PTGIR for angiogenesis in humans^35, 36^.

PGI2 is well known for its anti-proliferative action on vascular smooth muscle cells^11^. However, it may also stimulate the number of cells in the mouse embryo^37^ and the porcine trophoblast^23^. Here, we observed a stimulatory effect of iloprost on the proliferation of both pEETH and G1410 cells. Using pEETH cells, we demonstrated that a greater number of viable cells after the treatment with iloprost was detected only in the absence of a PTGIR antagonist. Therefore, PGI2 acts through its G protein-coupled receptor not only to modulate angiogenic gene expression in endometrial endothelial cells but also to stimulate cell proliferation. Further evidence for the relevant role of PGI2 was provided through the siRNA silencing approach; the knock-down of *PTGIS* resulted in decreased abundance of *KDR*, *FGFR2*, and *ANGPT2* transcripts and a lower number of viable cells as compared with respective controls. These results provide a clear evidence that an auto-/paracrine action of PGI2 is important for porcine endothelial cell transcription activity and cell proliferation. Similarly, proliferation of human endothelial progenitor cells was reduced after *PTGIS* silencing^38^.

Binding of PGI2 to PTGIR may activate various intracellular signaling pathways^16, 39^. Results of the present study revealed that PI3K and mTOR are involved in PGI2-stimulated endometrial endothelial cell proliferation, because the number of viable cells exposed to iloprost in the presence of LY294002 or rapamycin was lower compared with the number of cells exposed to iloprost alone. This observation coincides with previously reported importance of the activation of PI3K/Akt pathway for iloprost-induced development of porcine embryos^40^. As we showed here, blocking of adenylate cyclase activity in pEETH cells increased the number of viable cells both in the presence or absence of iloprost. Such result seems surprising because cAMP-PKA signaling pathway is responsible for the rapid response of vascular smooth muscle cells to PGI2^41^. Moreover, PGI2 stimulated cAMP formation in porcine endometrial epithelial and stromal cells^21^ and cAMP response element-binding protein was increased in porcine oocytes in response to iloprost^42^. Notably, activation of PKA in human endothelial cells inhibited cell migration in vitro and angiogenesis in vivo^43^. Nevertheless, present results showed that PI3K-mTOR pathway participates in PGI2-induced endometrial endothelial cell proliferation.

To gain more insight into pro-survival action of PGI2 on endothelial cells, we analyzed the distribution of cells in different phases of the cell cycle after the treatment with iloprost. The percentage of cells in S and G2/M phases was greater in the presence of iloprost as compared with control cells. It indicates that PGI2 promotes endothelial cell proliferation by accelerating cell division. This result differs from previously described an inhibitory effect of another analogue of PGI2, cicaprost, on S phase entry in human vascular smooth muscle cells^44^. To our knowledge, there is no data describing the effect of PGI2 on cell cycle progression in endothelial cells. However, an anti-apoptotic action of PGI2 in endothelial cells was reported^45^.

Another physiologically significant action of PGI2 tested here was its effect on cell migration. PGI2 has been reported as a stimulator of endothelial cell migration in humans^46, 47^. In fact, the wound healing assay allowed us to demonstrate that the treatment of endothelial cells with iloprost accelerated the gap closure. This effect was visible beginning from 42 h of culture. Most of studies however, describe cell migration as an activity occurring within 24 h^46–48^. Therefore, a lack of the difference between control and iloprost-treated cells within 24 h from seeding detected in the current research may indicate that in pigs PGI2 affects endothelial cell proliferation rather than their migration. Although the major advantage of the wound healing assay is that cells are monitored over time, the gap closure is a multi-step process involving spreading, migration, and proliferation of cells^49^. Indeed, the involvement of all these processes in the gap closure observed in the current experiment is possible.

The ability of endothelial cells to form capillary-like structures is commonly utilized to describe angiogenic activity^50, 51^. The present results demonstrate an inhibitory effect of a PGI2 analogue on the tube formation by porcine endothelial cells. The addition of iloprost to pEETH cells resulted in lower numbers of junctions and meshes and a smaller total meshes area as compared with the respective control. In human progenitor endothelial cells, blocking of PTGIR significantly weaker their angiogenic response^52^, while no effect of CAY10441 alone, except increased number of meshes, was detected in the present study. Most findings so far describing pro- or anti-angiogenic actions showed that examined factors either promoted or inhibited both cell proliferation and tube formation. Given the stimulatory effect of iloprost on endothelial cell proliferation detected in the present study its inhibitory action on pEETH cell sprouting was an unexpected result. To further verify this effect of PGI2 we used G1410 cell line which ability to form capillary-like structures has been previously reported^53^. As we showed here the effect of iloprost was time-dependent with decreased values of angiogenesis-related parameters measured after 3 and 4 h of treatment as compared with non-treated cells. However, after 6 h of incubation with iloprost the values of most parameters were not different from the control cells at this time point but were substantially increased as compared with values measured after 3 h of incubation with iloprost. It indicates that PGI2 delays the formation of capillary-like structures by porcine endothelial cells but does not completely block this process. The mechanisms underlying such an action of iloprost and the importance of this phenomenon are unknown. Most of studies involving the tube formation assay demonstrate results obtained at one selected time point. Thus, it is difficult to estimate whether such a phenomenon is specific to iloprost action on porcine endothelial cells only. Nevertheless, the present study showed that iloprost does not stimulate the formation of capillary-like structures in the pig uterine endometrium. Of interest, endogenous PGI2 is important for tube formation in melanoma and renal carcinoma endothelial cells but not in normal skin or kidney endothelial cells^36^. Therefore, the action of PGI2 on this process may rely on various physiological conditions what implies the necessity for further examinations of the role of PGI2-PTGIR signaling in the porcine endothelial cells. Furthermore, some mechanisms controlling endothelial tube formation and protecting from excessive angiogenesis must exist because several PGI2 analogues are used as therapeutic agents^10, 54^. Actually, several recent studies propose the existence of molecular mechanisms that when activated induce sprouting at the same time as they block cell proliferation and migration, and vice versa. This is due to the existence of intrinsic signaling feedback loops and cell cycle checkpoints that work in synchrony to maintain a balance between cell proliferation and sprouting^55, 56^. Further studies are required to identify possible mutually exclusive mechanisms related to angiogenic action of PGI2 in porcine endothelial cells.

## Conclusions

**Figure 8:**
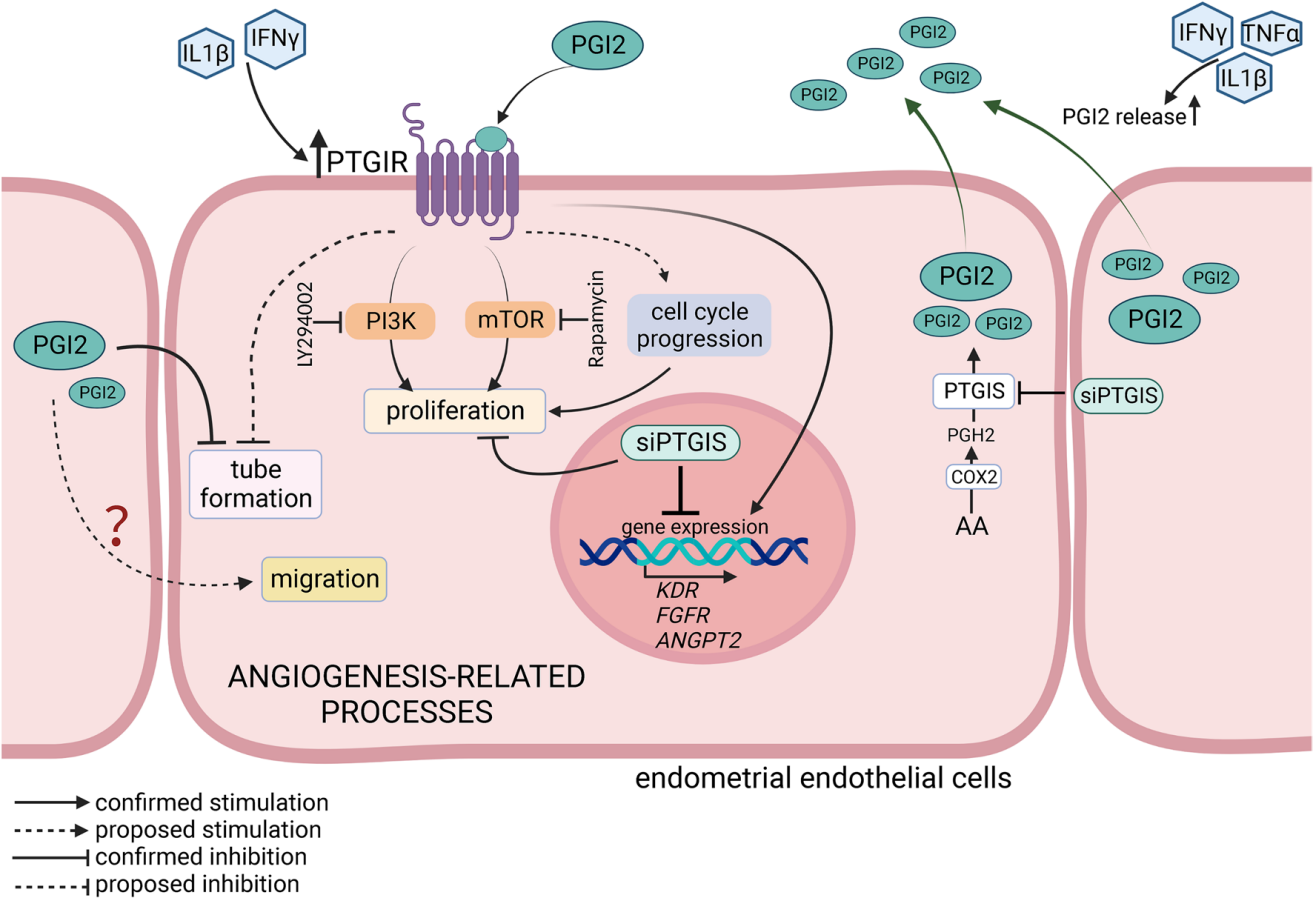
Schematic illustration of diverse PGI2 actions on endothelial cells of the porcine uterus. Increased amounts of cytokines, IL1β, IFNγ, and/or TNFα, present in the porcine uterus stimulate PGI2 secretion and PTGIR expression in endometrial endothelial cells. Abundant concentrations of PGI2, in turn, acting through PTGIR enhance the expression of angiogenesis-related genes, *KDR, FGFR2, ANGPT2,* and stimulates proliferation of endothelial cells. Accordingly, the knock-down of *PTGIS* inhibited the abundance of *KDR*, *FGFR2*, and *ANGPT2* transcripts and decreased the number of viable endothelial cells indicating auto- and/or paracrine actions of PGI2 on these cells. The PGI2-induced endothelial cell proliferation is mediated via the activation of PI3K and mTOR because specific inhibitors of these kinases (LY294002 and rapamycin, respectively) abolished the stimulatory effect of PGI2. Acting on porcine endothelial cells, PGI2 promotes cell cycle progression, while inhibits the formation of capillary-like structures. The effect of PGI2 on cell migration is also possible. Solid lines indicate stimulatory or inhibitory effects of PGI2 confirmed in the present study, dashed lines indicate possible pathways of PGI2 action. Prostacyclin (prostaglandin I2; PGI2), prostaglandin I2 receptor (PTGIR), arachidonic acid (AA; a substrate for PGI2 synthesis), cyclooxygenase 2 (COX2), prostaglandin H2 (PGH2), prostaglandin I2 synthase (PTGIS; terminal enzyme in PGI2 synthesis pathway), siRNA constructs targeting *PTGIS* (siPTGIS), interleukin 1β (IL1β), tumor necrosis factor α (TNFα), interferon γ (IFNγ), phosphoinositide 3-kinase (PI3K), mTOR kinase (mTOR), kinase insert domain receptor (KDR; vascular endothelial growth factor receptor), fibroblast growth factor receptor 2 (FGFR2), angiopoietin 2 (ANGPT2). Created with BioRender.com.

This study is the first comprehensive approach to describe an effect of PGI2 on angiogenesis-related events in the porcine endothelial cells. We demonstrated that cytokines, IL1β, IFNγ, and/or TNFα increased the secretion of PGI2 from endometrial endothelial cells and stimulated the expression of a PGI2 receptor, PTGIR, in these cells. PGI2, in turn, acting in an auto- and/or paracrine manner affected various endothelial cell functions (Fig. 8). In particular, PGI2 binding to PTGIR enhanced *KDR*, *FGFR2*, and *ANGPT2* mRNA expression and increased endothelial cell proliferation. The mitogenic effect of PGI2 on porcine endothelial cells was mediated by PI3K and mTOR signaling pathways and involved the stimulation of cell cycle progression. The formation of capillary-like structures by these cells however, was inhibited by PGI2. Moreover, the regulatory effect of PGI2 on the migration of endothelial cells should not be excluded but it requires further research. All these results suggest that the engagement of PGI2 in angiogenesis-related events in the porcine uterus is complex as pro- and anti-angiogenic effects are possible. Which physiological conditions trigger the switch between both PGI2 activities remain to be elucidated. In addition to presented here direct effects of PGI2 on endothelial cells, this prostanoid was shown to stimulate *VEGF* and *FGF2* expression in porcine endometrial stromal cells^21^. Therefore, the angiogenic activity of PGI2 in the endometrium may be the resultant of direct and indirect actions of PGI2 on endothelial and stromal cells, respectively. Taken together, PGI2 seems to be important for cyclical endometrial development in the porcine uterus.

## Materials and methods

### Ethics declarations

According to Directive 2010/63/EU of the European Parliament and of the Council of 22 September 2010 on the protection of animals used for scientific purposes, the ethical review and approval are not requested for this study because animal samples were collected post-mortem during regular slaughter process. All experiments were performed in compliance with the ARRIVE guidelines.

### Endometrial endothelial cell isolation and culture

Six crossbred gilts (Polish Landrace x Duroc) were slaughtered on days 6–8 of the estrous cycle to collect uteri (justification is provided in Supplementary Note). Uterine horns were transported on ice to the laboratory within one hour and subjected to the procedure of endothelial cell isolation. To this end, uterine horns of each gilt were cut into small fragments (10–15 cm long), washed with sterile phosphate-buffered saline (PBS; 137 mM NaCl, 27 mM KCl, 10 mM Na_2_HPO_4_, and 2 mM KH_2_PO_4_; pH 7.4) supplemented with antibiotics (100 IU/ml penicillin and 100 µg/ml streptomycin; Sigma-Aldrich, St. Louis, MO, USA), and cut open to separate the endometrium from the myometrium. The endometrial tissue was initially digested with 0.3% dispase (D4693; Sigma-Aldrich) in Hank’s balanced salt solution (H4891; Sigma-Aldrich; pH 7.4) at room temperature for 50 min with continuous stirring followed by a digestion using 0.06% collagenase I (C0130; Sigma-Aldrich) in Medium 199 (M2520; Sigma-Aldrich) supplemented with antibiotics and 0.5% bovine serum albumin (BSA; 81-003-3; Millipore, Kankakee, IL, USA) for 1.5 h at 37 °C. The cell suspension was filtered through a cell strainer with 100 µm pore size (352360; Corning, Corning, NY, USA) and centrifuged (800×*g*, 10 min). The obtained cells were washed with fresh Medium 199 supplemented with antibiotics and 5% NCS (N4637; Sigma-Aldrich). To separate endothelial cells from a mixture of stromal and epithelial cells, Dynabeads CD31 + (11155D; Invitrogen by Thermo Fisher Scientific, Waltham, MA, USA) were used according to the manufacturer’s protocol. Selected this way porcine endometrial endothelial (pEETH) cells were washed, suspended in MCDB-131 medium (M8537; Sigma-Aldrich) supplemented with antibiotics, 10% NCS, and 50 µg/mL endothelial cell growth supplement (ECGS; E2759; Sigma-Aldrich), and plated in culture flasks (Nunclon Delta T25 flasks; 130189; Thermo Fisher Scientific). Cells were passaged twice using 0.25% trypsin (25200; Gibco by Thermo Fisher Scientific) before being implemented in experiments (Experiments 1, 2, 4, and 8). For experiments, pEETH cells were suspended in MCDB-131 medium supplemented with antibiotics and 10% NCS, unless otherwise stated.

### G1410 cell culture

To further verify the relevance of PGI2 in angiogenesis-related processes, immortalized SUVECs (G1410 cell line) were used. Procedures of isolation, transfection, and characterization of this cell line (RRID: CVCL_F704) have been described earlier^57^. G1410 cell line has been previously used to examine angiogenesis in the porcine uterus^53^. G1410 cells were cultured in MCDB-131 medium supplemented with antibiotics, 10% NCS, and 50 µg/mL ECGS in Nunclon Delta T75 flasks (130190; Thermo Fisher Scientific). Cells were passaged twice using 0.25% trypsin before being implemented in experiments (Experiments 3, 5, 6, 7, and 8). For experiments, G1410 cells were suspended in MCDB-131 medium supplemented with antibiotics and 10% NCS, unless otherwise stated.

### Transfection of G1410 cells with siRNA

G1410 cells were seeded at a density of 0.1 × 10^6^ cells on 6-well plates (140675; Thermo Fisher Scientific) for mRNA and protein expression analysis or at a density of 0.1 × 10^5^ on 48-well plates (150687; Thermo Fisher Scientific) for proliferation assay. At about 50–60% of confluence, cells were transfected with 5 nM of siPTGIS (Sigma-Aldrich) or siNC (negative control, SIC001) diluted in serum-reduced Opti-MEM medium (11058021; Gibco by Thermo Fisher Scientific) using Lipofectamine RNAiMAX (13778; Invitrogen by Thermo Fisher Scientific) according to the manufacturer’s protocol. The siPTGIS sequences were GUGUUUAAAUACAACCGAU[dT][dT] (sense) and AUCGGUUGUAUUUAAACAC[dT][dT] (antisense). Control cells were incubated with Opti-MEM alone. Approximately 6 h after transfection, the medium was replaced with MCDB-131 medium supplemented with antibiotics and 1% NCS, and depending on the experiment, cells were cultured for additional 24 (mRNA analysis to confirm *PTGIS* silencing and for Experiment 3) or 48 (protein analysis to confirm *PTGIS* silencing and for Experiment 5) h.

For mRNA expression analysis, cells were washed with PBS, treated with a TRI Reagent (TR118; Molecular Research Center, Cincinnati, OH, USA), and stored at − 80 °C until total RNA extraction. For protein expression analysis, cells were washed with PBS, lysed in the cell extraction buffer (10 mM Tris–HCl, pH 7.4; 100 mM NaCl, 1 mM EDTA, 1% Triton X-100, 0.1% sodium dodecyl sulfate, 0.5% sodium deoxycholate) containing protease inhibitors (P8340; Sigma-Aldrich), and stored at − 40 °C until Western blot analysis.

### Experiment 1: Effect of cytokines on PGI2 synthesis and *PTGIR* expression in pEETH cells

To examine whether cytokines may affect PGI2 synthesis and its receptor expression, pEETH cells were seeded at a density of 0.5 × 10^6^ cells per well in 6-well culture plates. After reaching 90–95% confluency, cells were gently washed and treated with the basal medium (MCDB-131 medium supplemented with antibiotics and 0.1% BSA) or the basal medium containing the respective cytokine: interleukin 6 (IL6; I1395; Sigma-Aldrich), leukemia inhibitory factor (LIF; L5283; Sigma-Aldrich), IL1β (I9401; Sigma-Aldrich), TNFα (T6674; Sigma-Aldrich), or IFNγ (PSC4034; BioSource International Inc, Camarillo, CA, USA). All cytokines were used at the concentration of 10 ng/mL based on our previous studies^15, 21^. After 6 h, pEETH cells were washed with PBS, treated with Fenozol buffer (A&A Biotechnology, Gdansk, Poland), and stored at − 80 °C until total RNA extraction. Incubation media were collected and stored at − 40 °C until further analysis. The treatments were performed in duplicate in six separate experiments (gilts).

### Experiment 2: Effect of iloprost on the transcript abundance of genes related to angiogenesis and vascular function in pEETH cells

To examine whether PGI2 may affect the mRNA expression of factors involved in angiogenesis and vascular function, pEETH cells were seeded at a density of 0.5 × 10^6^ cells per well in 6-well culture plates. After reaching 90–95% confluency, cells were gently washed and pre-treated with the basal medium (MCDB-131 medium supplemented with antibiotics and 0.1% BSA) containing 0 or 10 µM CAY10441 (an antagonist of PTGIR; 10005186; Cayman Chemicals, Ann Arbor, MI, USA) for 30 min. Afterwards, the pre-treatment media were removed and discarded. For the next 24 h, cells were treated with the basal medium containing 0 or 0.1 µM iloprost (a PGI2 analogue; 18215; Cayman Chemicals) in the presence or absence of CAY10441 (10 µM). Concentrations of iloprost and CAY10441 were chosen based on our previous studies^15, 21, 23^. Then, pEETH cells were washed with PBS, treated with Fenozol buffer, and stored at − 80 °C until total RNA extraction. All treatments were performed in duplicate in six separate experiments (gilts).

### Experiment 3: Effect of *PTGIS* silencing on the transcript abundance of selected genes related to angiogenesis and vascular function in G1410 cells

To further verify the importance of PGI2 for the mRNA expression of angiogenesis-related factors, G1410 cells were seeded at a density of 0.1 × 10^6^ cells on 6-well plates and subjected to the transfection procedure as described above. After 24 h post-transfection culture, cells were washed with PBS, treated with a TRI Reagent, and stored at − 80 °C until total RNA extraction. The treatments were performed in duplicate using cells from four different passages.

### Experiment 4: Effect of iloprost on pEETH cell proliferation

To determine whether PGI2 may influence endothelial cell proliferation, pEETH cells were seeded at a density of 6 × 10^3^ cells per well in 96-well culture plates (165306; Thermo Fisher Scientific). After reaching 50–55% confluency, cells were gently washed and serum starved for 16 h. Then, cells were pre-treated with the basal medium (MCDB-131 medium supplemented with antibiotics and 1% NCS) containing 0 or 10 µM of CAY10441 for 30 min. Subsequently, cells were treated for 24 h with the basal medium containing iloprost (0, 0.1, or 1 µM) in the presence or absence of CAY10441 (10 µM).

To identify intracellular kinases that potentially participate in the action of PGI2 on cell proliferation, pEETH cells were pre-treated with the basal medium (MCDB-131 medium supplemented with antibiotics and 1% NCS) containing no inhibitors (control) or the respective inhibitor: SQ 22536 (an inhibitor of adenylate cyclase; 568500; Sigma-Aldrich; 10 µM), LY294002 (an inhibitor of phosphatidylinositol 3-kinase [PI3K]; 9901; Cell Signaling Technology, Danvers, MA, USA; 20 µM), U0126 (an inhibitor of MEK1 and MEK2; 9903; Cell Signaling Technology; 10 µM), or rapamycin (an inhibitor of mTOR kinase; R8781; Sigma-Aldrich; 20 nM) for 60 min (Supplementary Note). Subsequently, cells were treated for 24 or 48 h with the basal medium containing 0 or 0.1 µM of iloprost in the presence or absence of the respective inhibitor.

For both proliferation assays, 10% NCS was used as a positive control. After incubation, 0.2% crystal violet was used to stain viable cells and the absorbance was measured at 550 nm wavelength. The treatments were performed in triplicate in six separate experiments (gilts).

### Experiment 5: Effect of iloprost or *PTGIS* silencing on G1410 cell proliferation

To verify the effect of PGI2 on proliferation of endothelial cells, G1410 cells were seeded in 48-well culture plates at a density of 0.1 × 10^5^ cells per well. After reaching 50–60% confluency, the media were discarded and G1410 cells were treated with 0 or 0.1 µM of iloprost in MCDB-131 supplemented with antibiotics and 1% NCS, or cells were subjected to the transfection procedure (as described above). After 48 h of the treatment with iloprost or the post-transfection culture, CellTiter 96 Aqueous One Solution Reagent (G3580; Promega, Madison, WI, USA) was added into each well (20 µL per well) for 2 h and the absorbance was measured at 490 nm wavelength. All treatments were performed in triplicates using G1410 cells from three different passages.

### Experiment 6: Effect of iloprost on cell cycle progression of G1410 cells

To examine the effect of PGI2 on cell cycle progression, FxCycle Violet Stain (F10347; Invitrogen by Thermo Fisher Scientific) was used according to the manufacturer’s instruction. Briefly, G1410 cells were seeded at a density of 1 × 10^6^ cells per T25 culture flask. After reaching 80–90% confluence, cells were exposed to 0 or 0.1 µM of iloprost in MCDB-131 medium supplemented with antibiotics and 1% NCS for 24 h. Then, cells were harvested using 0.25% trypsin and centrifuged (800×*g*, 7 min, 8 °C). The supernatant was discarded and MCDB-131 medium was added to the cell pellet. Subsequently, 1 × 10^6^ cells were fixed in 1% (vol/vol) formaldehyde (252549; Sigma-Aldrich) in MCDB-131 medium for 10 min and quenched by 2.5 M glycine for 5 min. The cell suspension was washed in ice-cold PBS and centrifuged (800×*g*, 7 min, 8 °C). Then, cells were suspended in 1 mL of ice-cold PBS and 1 μL of FxCycle Violet stain was added to each sample. Samples were incubated for 30 min at room temperature, protected from light. Flow cytometric analysis of DNA content distribution was performed at 375 nm excitation with a 450/40 bandpass filter. Each treatment was performed using G1410 cells from three different passages.

### Experiment 7: Effect of iloprost on G1410 cell migration; a wound-healing assay

To determine whether PGI2 may affect migration of endothelial cells, the Culture-Insert 2 Well system (81176; ibidi GmbH, Gräfelfing, Germany) was used according to the manufacturer’s protocol. Briefly, G1410 cells were seeded at a density of 2 × 10^4^ cells per chamber in the Culture-Insert 2 Well placed in 24-well culture plates (142475; Thermo Fisher Scientific). After reaching 100% confluency, the Culture-Inserts were removed and cells were washed with PBS to remove non-adherent cells. MCDB-131 medium supplemented with antibiotics and 1% NCS containing 0 or 0.1 µM of iloprost was added into respective chambers. Cells were monitored for 84 h and photographed every 2 h using the Zeiss Axio Observer System with software ZEN Blue 2.5 (Carl Zeiss Microscopy GmbH, Jena, Germany). The area of the gap was measured using the Fiji software with the MRI Wound Healing Size Tool^48^. Data are expressed as the percentage of wound closure. The treatments were performed using G1410 cells from four different passages.

### Experiment 8: Effect of iloprost on the formation of capillary-like structures by pEETH and G1410 cells

To determine whether PGI2 may affect the formation of capillary-like structures by pEETH and G1410 cells, µ-Slide angiogenesis plates (81506; ibidi GmbH, Gräfelfing, Germany) covered with growth factor reduced Matrigel^®^ Basement Membrane Matrix (354230; Corning, Bedford, MA, USA) were used. To achieve this, pEETH cells were pre-treated with the basal medium (MCDB-131 medium supplemented with antibiotics) containing 0 or 10 µM of CAY10441 for 30 min. Subsequently, pEETH cells were suspended in the basal medium containing 0 or 0.1 µM of iloprost in the presence or absence of CAY10441. G1410 cells were treated with 0 or 0.1 µM of iloprost in the basal medium. Both pEETH and G1410 cells were plated at the concentration of 1 × 10^4^ cells per well onto µ-Slide angiogenesis plates and incubated for 6 h at 37 °C in a humidified atmosphere of 95% air and 5% CO_2_ in a Zeiss Axio Observer System. Each well was photographed at 30-min intervals. The potential of pEETH and G1410 cells to form capillary-like structures characteristic for angiogenesis^50, 51^ was analyzed using ImageJ software with angiogenesis plug-in. The following structures were analyzed: the number of extremes, the number of nodes, the number of junctions, the number of meshes, total meshes area, the number of segments, total segments length, and the number of branches. This experiment was performed in triplicate in three separate experiments (gilts; for pEETH cells) or cells from three different passages (for G1410 cells).

### Immunofluorescent staining of pEETH and G1410 cells

To examine the presence of vWF (a marker of endothelial cells) and PTGIR proteins, pEETH and G1410 cells were cultured in Cell Imaging Coverglasses (0030742028; Eppendorf, Hamburg, Germany), fixed in 4% paraformaldehyde in PBS, and subjected to the staining procedure^58^. The incubation with rabbit polyclonal anti-PTGIR receptor (10005518; Cayman Chemical; dilution 1:50) or rabbit polyclonal anti-vWF (A0082; Dako Cytomation, Glostrup, Denmark; dilution 1:50) antibodies was performed overnight at 4 °C. Negative control staining was accomplished by replacing the primary antibody with the rabbit IgG negative control (I-1000; Vector Laboratories, Inc., Burlingame, CA, USA). Afterwards, all sections were incubated with CY^3^-conjugated donkey anti-rabbit IgG (dilution 1:1000; 711-165-152; Jackson ImmunoResearch). Actin filaments and nuclei were visualized using CytoPainter Phalloidin-iFluor 488 Reagent (ab176753; abcam, Cambridge, UK) and diamidino-2-phenylindole (DAPI; Vector Laboratories), respectively. Slides were mounted in a Vectashield Mounting Medium (Vector Laboratories) and photographed using an Olympus digital camera.

### Total RNA isolation and real-time PCR

Total RNA from pEETH (Experiments 1 and 2) and G1410 (*PTGIS* knock-down confirmation and Experiment 3) cells was extracted using a Total RNA Prep Plus kit (031-100; A&A Biotechnology) and RNeasy Mini Kit (74104; Qiagen, Valencia, CA, USA), respectively, according to the manufacturers’ protocols. Samples were treated with DNase I (AMPD1; Sigma-Aldrich) and reverse transcribed using a High Capacity cDNA Reverse Transcription Kit (Applied Biosystems by Thermo Fisher Scientific), as described earlier^15^.

Diluted cDNA from RT-PCR was used to determine relative mRNA abundance of selected genes with an ABI Viia7 Sequence Detection System (Life Technologies Inc., Carlsbad, CA, USA). In order to determine the mRNA expression of *PTGIS, PTGIR, VEGFA, KDR, FLT1, FGF2, FGFR2*, *ICAM1, ANGPT1, ANGPT2, TIE1, TEK, ACTB*, *HPRT1*, and *GAPDH*, 15 (for pEETH cells) or 20 (for G1410 cells) ng of cDNA was amplified using TaqMan Gene Expression Assays (Applied Biosystems by Thermo Fisher Scientific). All abbreviations of the examined genes, their full names, and the ID numbers of TaqMan probes are outlined in Supplementary Table S1. Each PCR reaction (10 µL) was carried out in duplicates in 384-well plates using the following conditions: initial denaturation for 10 min at 95 °C, followed by 40 cycles of 15 s denaturation at 95 °C and then 60 s of annealing at 60 °C. To test for genomic DNA contamination, the control reactions in the absence of reverse transcriptase were performed. For both RT-PCR and qPCR, no template controls with nuclease-free water were conducted to check for possible reagent contamination. Data from Real-time PCR were analyzed using the PCR Miner algorithm^59^ (pEETH) or the relative mRNA expression was calculated as 2^−∆Ct^ = 2^−(Ct target gene–Ct housekeeping gene)^^60^ (G1410 cells). NormFinder software^61^ was used to select the most stable reference genes among *GAPDH*, *HPRT1*, and *ACTB*. All expression data for each target gene were normalized against geometric averaging of *ACTB* and *HPRT1* (for pEETH cells) and of *GAPDH* and *HPRT1* (for G1410 cells).

### Western blot

Equal amounts (10 µg) of total protein extracted from G1410 cell samples were dissolved in SDS gel-loading buffer (50 mM Tris–HCl, pH 6.8; 4% SDS, 20% glycerol, and 2% β-mercaptoethanol), heated to 95 °C for 5 min, and separated on 10% SDS-PAGE. Proteins were electroblotted onto 0.45 µm pore size polyvinylidene difluoride membrane in transfer buffer (20 mM Tris–HCl, pH 8.2; 100 mM glycine, 20% methanol). Nonspecific binding sites were blocked with 5% nonfat dry milk in TBS-T (Tris-buffered saline, pH 7.4; 0.1% Tween-20) at room temperature for 1.5 h. Afterwards, membranes were incubated overnight with the anti-PTGIS polyclonal antibody (160640; Cayman Chemical; dilution 1:200), washed with TBS-T and incubated for 1 h with the anti-rabbit IgG-alkaline phosphatase antibody (A8024; Sigma-Aldrich; 1:20,000 dilution). Immune complexes were visualized using a standard alkaline phosphatase visualization procedure. As internal control for protein loading, the rabbit polyclonal to GAPDH antibody (ab9485; abcam; dilution 1:2000) was used.

### Enzyme-linked immunosorbent assay (ELISA) of PGI2 metabolite

Concentrations of 6-keto PGF1α (a stable metabolite of PGI2) in incubation media collected during Experiment 1 were determined using an ELISA kit (515211; Cayman Chemical), according to the manufacturer’s protocol. The sensitivity of the assay was 1.6 pg/mL, and the intra-assay coefficient of variation was 6.5%.

### Statistical analyses

Statistical analyses were conducted using GraphPad PRISM v. 9.3.1. (GraphPad Software, Inc., San Diego, CA, USA). To analyze the effect of cytokines on *PTGIS* and *PTGIR* mRNA expression and 6-keto PGF1α concentration, one-way ANOVA followed by the Bonferroni multiple comparison test was used. To test the effect of PGI2 on (1) the abundance of angiogenesis-related transcripts in pEETH cells, (2) pEETH cell proliferation, (3) G1410 cell cycle progression, (4) G1410 cell migration, and (5) capillary-like tube formation by pEETH and/or G1410 cells, two-way ANOVA followed by Bonferroni post hoc test was conducted. To examine the effect of (1) NCS on pEETH cell proliferation, (2) iloprost on G1410 cell proliferation, or (3) *PTGIS* knock-down on G1410 cell proliferation and mRNA and protein expression, a paired *t*-test was used. Data from Experiment 6 were log transformed due to variability between experimental sets. All numerical data are presented as means ± SEM. Means were considered to be statistically different at *p* ≤ 0.05.

### Supplementary Information

Supplementary Information.

## Data Availability

The datasets generated and analyzed during the current study are available from the corresponding author on reasonable request.
