# Supplementary material for: Diverse effects of prostacyclin on angiogenesis-related processes in the porcine endometrium
Source: Sci Rep. 2023 Aug 29;13:14133. doi: 10.1038/s41598-023-41197-z (PMC10465533; doi:10.1038/s41598-023-41197-z)
Supplement: Supplementary file 1 — Supplementary Information. [file 41598_2023_41197_MOESM1_ESM.pdf]

Diverse effects of prostacyclin on angiogenesis-related processes in the porcine endometrium

Magdalena Szymanska and Agnieszka Blitek*

Institute of Animal Reproduction and Food Research of the Polish Academy of Sciences, Tuwima 10, 10-748 Olsztyn, Poland

*Corresponding author: a.blitek@pan.olsztyn.pl

pEETH cells

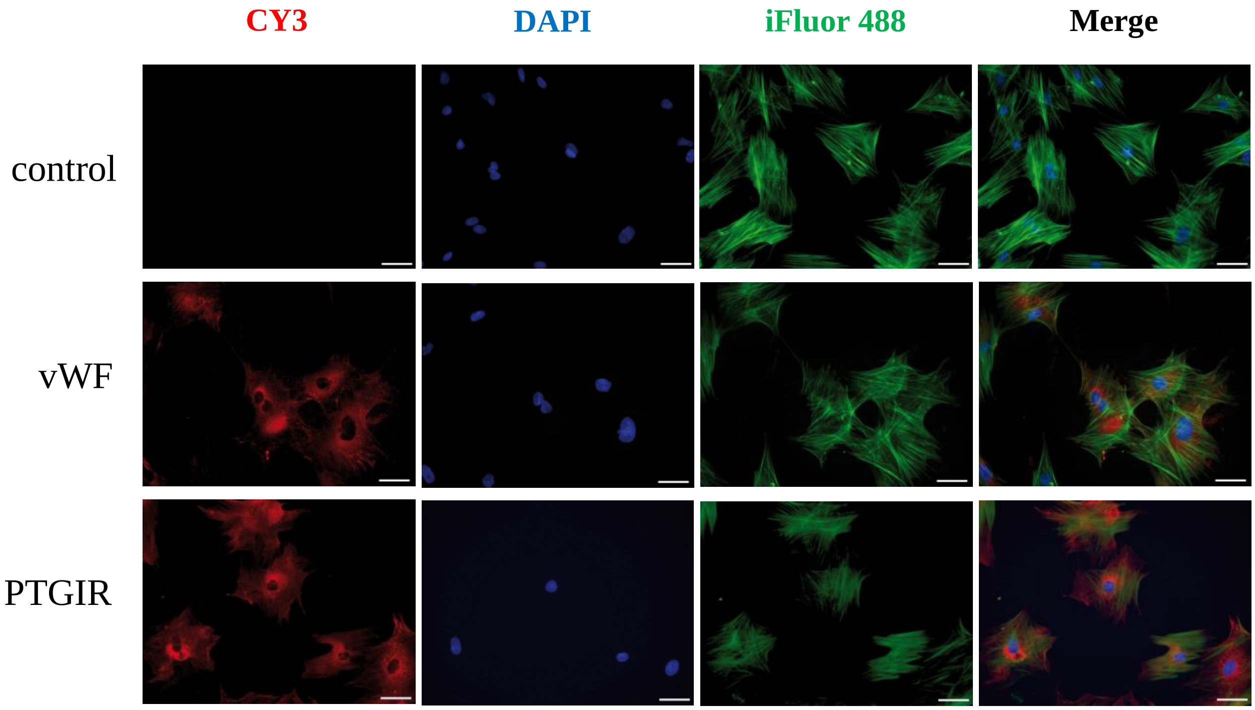

Supplementary Fig. S1. The expression of von Willebrand Factor (vWF; a marker of endothelial cells) and prostaglandin I2 receptor (PTGIR) proteins in porcine endometrial endothelial (pEETH) cells. Cells were counterstained with diamidino-2-phenylindole (DAPI) and CytoPainter Phalloidin-iFluor 488 Reagent (iFluor 488) to visualize nuclei and actin filaments, respectively. Negative control was accomplished by replacing the primary antibody with rabbit IgG negative control; scale bars 50 µm.

G1410 cells

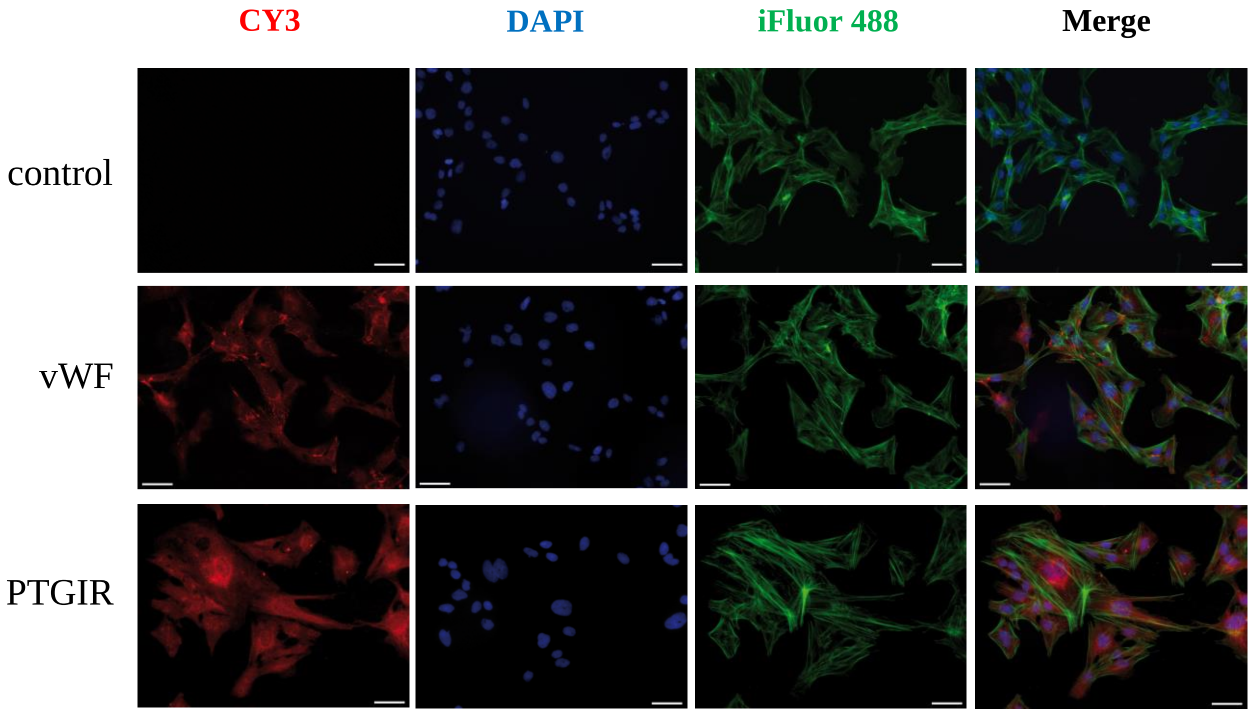

Supplementary Fig. S2. The expression of von Willebrand Factor (vWF; a marker of endothelial cells) and prostaglandin I2 receptor (PTGIR) proteins in immortalized swine umbilical vein endothelial cells (G1410 cell line). Cells were counterstained with diamidino-2-phenylindole (DAPI) and CytoPainter Phalloidin-iFluor 488 Reagent (iFluor 488) to visualize nuclei and actin filaments, respectively. Negative control was accomplished by replacing the primary antibody with rabbit IgG negative control; scale bars 50 µm.

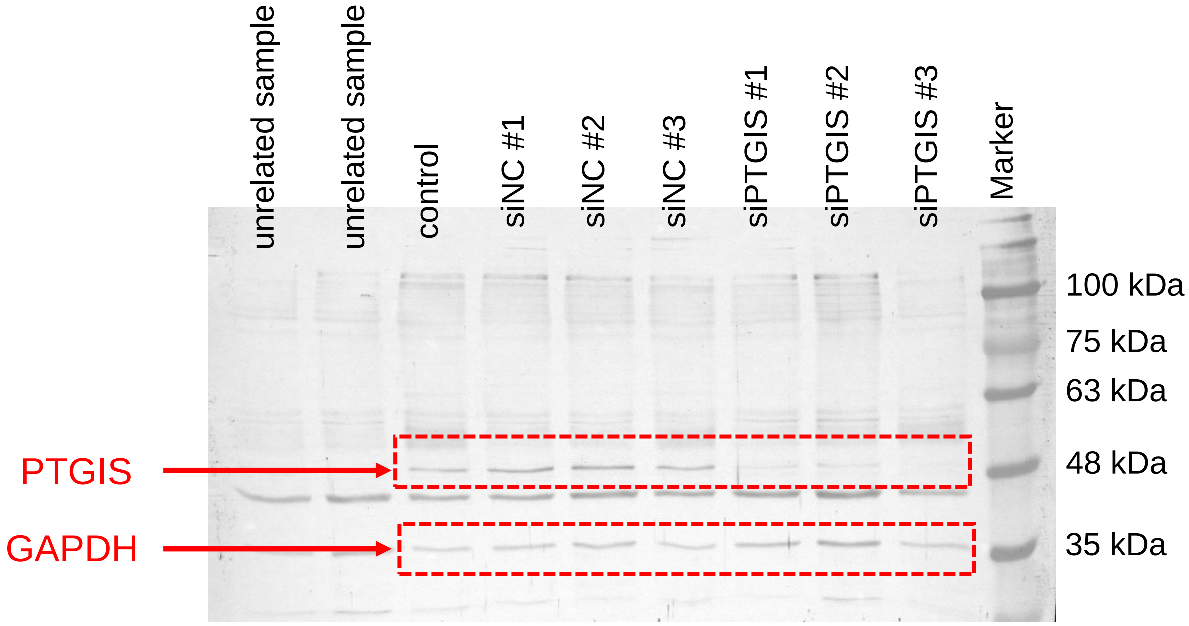

Supplementary Fig. S3. Western blot results presenting prostaglandin I2 synthase (PTGIS) and glyceraldehyde-3-phosphate dehydrogenase (GAPDH) protein expression in G1410 cells transfected with siRNA targeting *PTGIS* (siPTGIS #1, #2, #3) or with scrambled siRNA (siNC #1, #2, #3) or incubated with Opti-MEM alone (control). The red dashed line shows fragments of the blot presented in Fig. 3.

Supplementary Note

Justification for choosing days 6 to 8 of the estrous cycle to isolate endometrial endothelial cells

The uterine blood supply in pigs is the lowest on days 10 to 12 of the estrous cycle [1]. The volume of blood flowing into the uterus falls to 30-40% of that observed during estrus [1] but the uterine weight is about 50% greater than that measured in the early luteal phase [2]. Day 12 of the estrous cycle is the critical threshold in uterine circulation status, after which regressive changes in the endometrium are initiated [2]. Therefore, days 6 to 8 were chosen as a period of normally developing functional endometrium which may be a source of sufficient number of endothelial cells.

Justification for choosing applied concentrations of kinase inhibitors

Concentrations of kinase inhibitors were selected based on the available literature:

- SQ 22536; the dose of 10 μM was reported as effective in experiments on cultured cells, including brain capillary endothelial cells [3] and coronary smooth muscle cells [4];
- LY294002 ; the dose of 20 μM was effective in blocking VEGF-stimulated [5] and IGF-I-stimulated [6] proliferation of porcine trophoblast cells;
- U0126; the dose of 10 μM was effective in blocking ERK phosphorylation in COS-7 cells [7], human hepatocytes [8], or human follicles [9];
- Rapamycin; the dose of 20 nM was effective in blocking VEGF-stimulated [5] and IGF-I-stimulated [6] proliferation of porcine trophoblast cells.

References for Supplementary Note:

[1] Ford, S.P. & Christenson, R.K. Blood flow to uteri od sows during the estrous cycle and early pregnancy: local effect of the conceptus on the uterine blood supply. *Biol. Reprod.* **21**, 617-624 (1979).

[2] Krzymowski, T. & Stefanczyk-Krzymowska, S. The oestrous cycle and early pregnancy – a new concept of local endocrine regulation. *Vet. J.* **168**, 285-296 (2004).

[3] Dohgu, S. *et al.* Cyclosporin A induces hyperpermeability of the blood-brain barrier by inhibiting autocrine adrenomedullin-mediated up-regulation of endothelial barrier function. *Eur. J. Pharmacol.* **644**, 5-9 (2010).

[4] Son, Y.K. *et al.* Protein kinase A-dependent activation of inward rectifier potassium channels by adenosine in rabbit coronary smooth muscle cells. *Biochem. Biophys. Res. Commun.* **337**, 1145-1152 (2005).

[5] Jeong, W., Kim, J., Bazer, F.W. & Song, W. Stimulatory effect of vascular endothelial growth factor on proliferation and migration of porcine trophectoderm cells and their regulation by the phosphatidylinositol-3-kinase-AKT and mitogen-activated protein kinase cell signaling pathways. *Biol. Reprod.* **90**, 1-10 (2014).

[6] Jeong, W., Song, G., Bazer, F.W. & Kim, J. Insulin-like growth factor I induces proliferation and migration of porcine trophectoderm cells through multiple signaling pathways, including protooncogenic protein kinase 1 and mitogen-activated protein kinase. *Mol. Cell. Endocrinol.* **384**, 175-184 (2014).

[7] Favata, M.F. *et al.* Identification of a novel inhibitor of mitogen-activated protein kinase kinase. *J. Biol. Chem.* **273**, 18623-18632 (1998).

[8] Smutny, T. *et al.* U0126, a mitogen-activated protein kinase kinase 1 and 2 (MEK1 and 2) inhibitor, selectivelu up-regulates main isoforms of CYP3A family via a pregnane X receptor (PXR) in HepG2 cells. *Arch. Toxicol.* **88**, 2243-2259 (2014).

[9] Zhao, Y. *et al.* MAPK3/1 participates in the activation of primordial follicles through mTORC1-KITL signaling. *J. Cell. Physiol.* **233**, 226-237 (2017).

Supplementary Table S1. Full names of genes and the ID numbers of TaqMan probes used to examine relative mRNA abundance in porcine endometrial endothelial (pEETH) cells and/or immortalized swine umbilical vein endothelial cells (G1410 cell line).

Abbreviation	Gene name	ID of TaqMan probe
PTGIS	prostaglandin I2 synthase	Ss03374149_m1
PTGIR	prostaglandin I2 receptor	–[1]
VEGFA	vascular endothelial growth factor A	Ss03393993_m1
KDR	kinase insert domain receptor	Ss03375683_u1
FLT1	fms related receptor tyrosine kinase 1	Ss03375679_u1
FGF2	fibroblast growth factor 2	Ss03375809_u1
FGFR2	fibroblast growth factor receptor 2	Ss03389140_m1
ICAM1	intercellular adhesion molecule 1	Ss03392385_m1
ANGPT1	angiopoietin1	Ss03391079_m1
ANGPT2	angiopoietin 2	Ss03392362_m1
TIE1	tyrosine kinase with immunoglobulin like and EGF like domains 1	Ss03373579_g1
TEK	TEK receptor tyrosine kinase	Ss03373354_m1
ACTB	actin beta	Ss03376081_u1
HPRT1	hypoxanthine phosphoribosyltransferase 1	Ss03388274_m1
GAPDH	glyceraldehyde-3-phosphate dehydrogenase	Ss03375435_u1

[1]Designed by Applied Biosystem (Thermo Fisher Scientific); GeneBank accession no. NC_010448.3.
